# Rerouting the Dissection of the Infratemporal and Submandibular Regions

**DOI:** 10.7759/cureus.15227

**Published:** 2021-05-25

**Authors:** Deepika Poonia, Dinesh Kumar, Shahid B Rangrej

**Affiliations:** 1 Anatomy, Maulana Azad Medical College, New Delhi, IND; 2 Anatomy, Saint James School of Medicine, Kingstown, VCT

**Keywords:** cadaveric dissection, digastric muscle, hypoglossal nerve, lingual nerve, mylohyoid, temporalis muscle, masseter muscle, lingual duct, gland, genioglossus

## Abstract

Introduction

Teaching and learning in anatomy are necessarily dependent on cadaveric dissection. Skillful dissection is the tool which helps in proper visualization of structures in a cadaver. Proper understanding about the course of lingual nerve, hypoglossal nerve, nerve to mylohyoid, and relations between structures present in infratemporal and submandibular regions is important for medical students. The aim of this study is to describe a modified technique of dissection and evaluate medical students' and teachers’ response to this approach.

Methods

The comparative observational study was conducted bilaterally on six adult cadavers. We compared the method of dissection given in standard textbooks with the modified method introduced. The validity and reliability of the newer method of dissection for teaching purpose was assessed by first-year undergraduate medical students using a questionnaire-based tool and feedback from postgraduate students and senior residents.

Results

The modified method was described as less time consuming, easy to perform, and allowed extensive exploration of the structures in the infratemporal and submandibular regions.

Conclusions

Proper understanding of the course and relations between structures present in infratemporal and submandibular regions is important for medical students.The modified approach to infratemporal and submandibular regions will facilitate better understanding of the human anatomy.

## Introduction

Human anatomy is considered the cornerstone of medical education [1]. At any stage of learning anatomy in medical schools, cadaveric dissection cannot be overemphasized as it is the most authentic and far superior method to any other available learning resource for developing interest in and understanding of anatomy [2]. Anatomical comprehension of infratemporal fossa (ITF) and submandibular region (SMR) is challenging for students as well as teachers due to the complex relationships between various structures present in this highly compact region. Understanding the anatomy of ITF is pivotal for surgeons aiming for the lateral skull base approach [3,4,5]. Variations in the course and relations of submandibular duct, lingual nerve, and hypoglossal nerve are commonly reported. A better understanding of normal anatomy of peri-mandibular region is pertinent for reconstructive surgeons to avoid intra-operative complications [6,7]. Difficulty in understanding the anatomy of this region is further compounded by the current documented methods of dissection, which are tedious, difficult, and time consuming. To resolve this issue, the authors aimed to design a modified dissection method to expose the contents of ITF and SMR.

## Materials and methods

The cadaveric study was conducted in the Department of Anatomy, Maulana Azad Medical College, Delhi, India. Cadavers without any gross pathology and deformity over head and neck region were included in the study. The dissection was performed on 12 sides of six formalin-fixed adult cadavers donated to the Department of Anatomy for study and research purposes. On all the six cadavers, ITF and SMR were dissected on one side of the face using the steps described in *Cunningham’s Manual of Practical Anatomy*, fifteenth edition, as part of routine dissection [8], and other side of face was dissected by the modified method. On each cadaver, both the sides of the head and neck were dissected by a single resident doctor and a postgraduate student. Photographs were taken after every step of dissection.

The current study is a research on the comparison of effectiveness of dissection techniques, as part of undergraduate teaching program and hence exempt from Institutional Ethical Committee review as per existing institutional guidelines. The non-probability sampling method (convenience sampling method) was used to select 25 undergraduate students to participate in the study. The undergraduate students were shown the underlying structures of the dissected ITF and SMR. The simple statistical method was used for analysis of responses obtained from the undergraduate students.

Steps of modified dissection technique

Step 1

After completion of parotid gland dissection, following the steps described by Romanes [8], zygomatic arch was cut. Masseter muscle exposed and was cut close to its attachment on mandible.

Step 2

**Figure 1:**
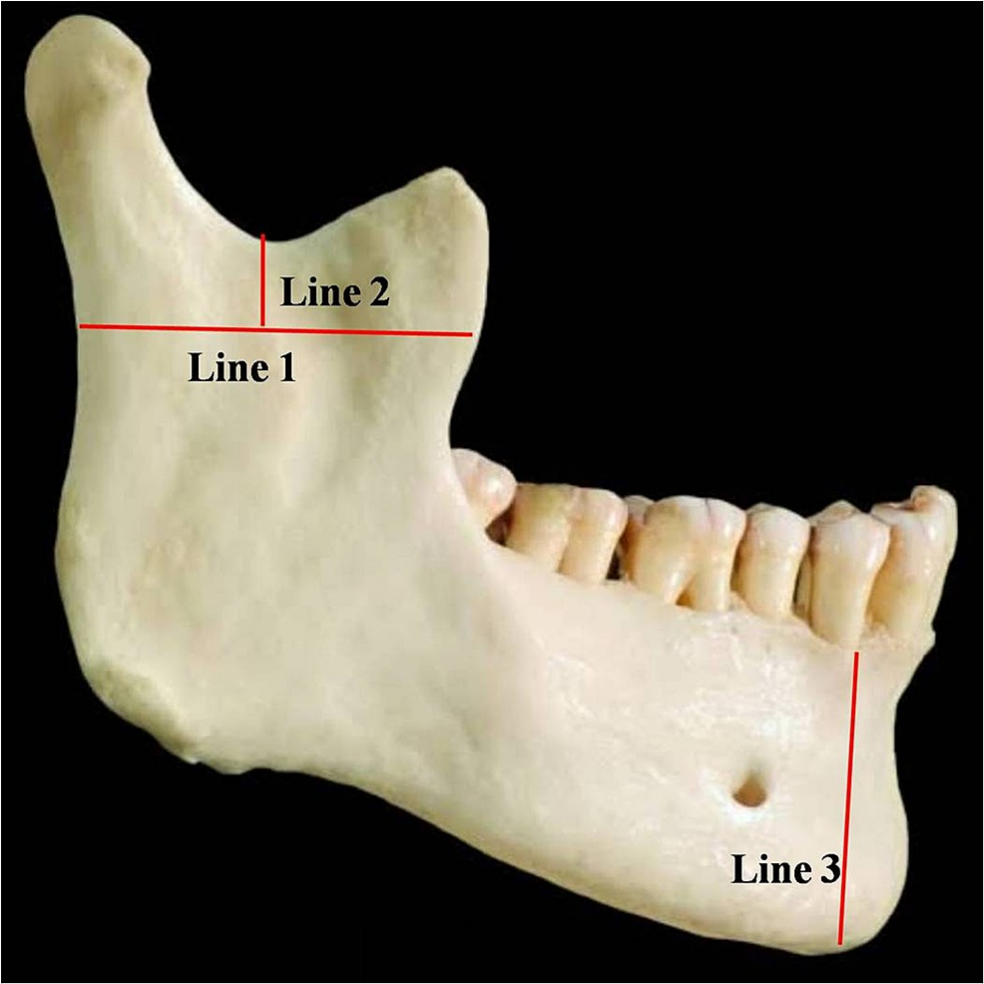
Lines along which the mandible was cut.

The mandible bone was cut along two lines, i.e., Line 1 and Line 2 (Figure 1).

Step 3

The coronoid process along with the fibers of temporalis muscle (TM) was lifted anteriorly away from ramus of mandible and turned cranially. Fibers of lateral pterygoid muscle were seen placed transversely. Maxillary artery (first and second part) was identified and its branches were traced. Students were shown inferior alveolar nerve and vessels (IAN, IAV) entering into the mandibular foramen, which were then cut near the foramen.

Step 4

**Figure 2:**
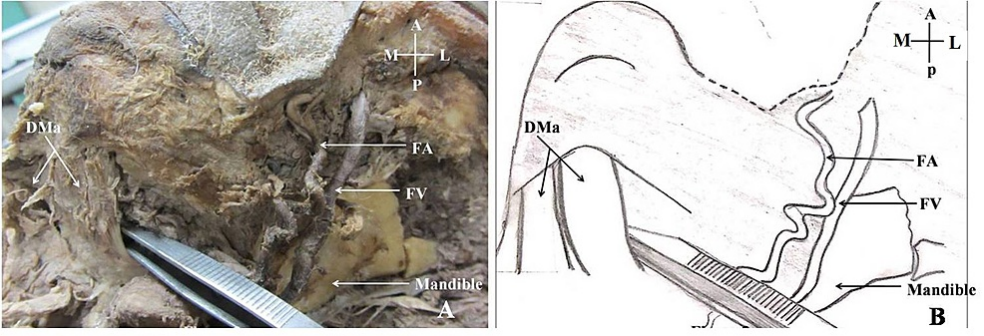
Lateral border of the left-side anterior belly of the digastric muscle cleared to place a paramedian cut on the mandible. A: Cadaveric view of the infratemporal fossa and submandibular region; B: Schematic representation of the cadaveric view; DMa: Anterior belly of the digastric muscle; FA: Facial artery; FV: Facial vein; A: Anterior; P: Posterior; M: Medial; L: Lateral.

The body of mandible was cut along the Line 3 near the lateral border of digastric muscle (Figure 2).

**Figure 3:**
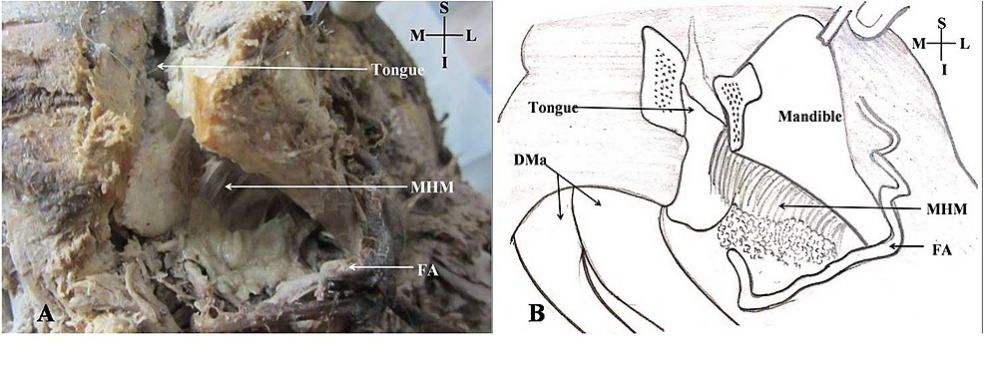
Mandible pulled to expose the left-side mylohyoid muscle. A: Cadaveric view of the submandibular region; B: Schematic representation of the cadaveric view; MHM: Mylohyoid muscle; S: Superior; I: Inferior; M: Medial; L: Lateral; DMa: Anterior belly of the digastric muscle; FA: Facial artery.

The mylohyoid muscle was identified from below, i.e., its superficial surface was cut near its attachment on mylohyoid line using scalpel. The genioglossus, geniohyoid, and anterior belly of the digastric muscles were left intact (Figure 3).

**Figure 4:**
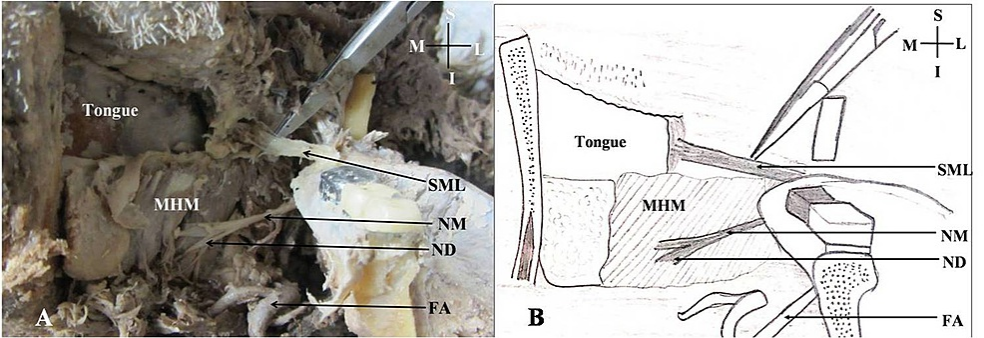
Mandible pulled on the left side to expose the sphenomandibular ligament and the nerve to the mylohyoid muscle. A: Cadaveric view of the submandibular region; B: Schematic representation of the cadaveric view; SML: Sphenomandibular ligament; NM: Nerve to the mylohyoid muscle; ND: Nerve to the anterior belly of the digastric muscle; S: Superior; I: Inferior; M: Medial; L: Lateral; FA: Facial artery; MHM: Mylohyoid muscle.

The sphenomandibular ligament was palpated and then incised near the lingula. Hence, the body of mandible was separated from the underlying structures progressively from the anterior to the posterior side (Figure 4).

**Figure 5:**
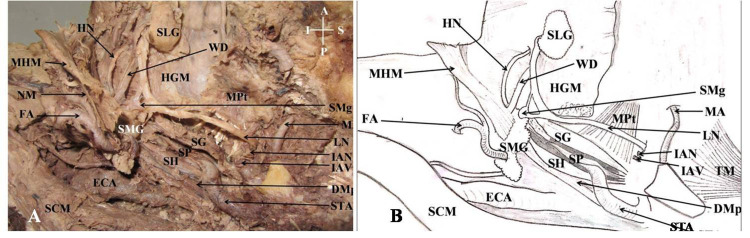
Structures on the left side, lying in superficial relation to the hyoglossus, submandibular ganglion, and muscles of the styloid apparatus. A: Cadaveric view of the infratemporal fossa and submandibular region; B: Schematic representation of the cadaveric view; ECA: External carotid artery; SCM: Sternocleidomastoid muscle; DMp: Posterior belly of Digastric muscle; MPt: Medial pterygoid muscle; MA: Maxillary artery;  STA: Superficial temporal artery; HGM: Hyoglossus muscle; HN: Hypoglossal nerve; MHM: Mylohoid muscle; TM: Temporalis muscle; IAN: Inferior alveolar nerve; IAV: Inferior alveolar vessels; LN: Lingual nerve; SMG: Submandibular gland; SLG: Sublingual gland; MPt: Medial pterygoid muscle; SG: Styloglossus muscle; SP: Stylopharyngeus muscle; SH: Stylohyoid muscle; WD: Wharton duct; SMg: Submandibular ganglion.

The body and ramus of mandible were removed, i.e., a hemi-mandibulectomy was performed. The muscles were then identified from the anterior and posterior sides as genioglossus (appeared fan-shaped), geniohyoid (strap-like), anterior belly of digastric (continuous with the posterior belly of digastric and placed superficial to geniohyoid muscle), and mylohyoid muscle. The mylohyoid muscle was reverted to expose the deep part of the submandibular gland (SMG) and the sublingual gland (SLG). The course of the lingual nerve in the submandibular region deep to the mylohyoid muscle was also exposed. The relation of the lingual nerve (LN) with the Wharton duct (WN) and the sublingual gland along with the submandibular ganglion (SMg) was then cleared. Hypoglossal nerve (HN) was seen deep to mylohyoid muscle. Deep to hypoglossal nerve, glossopharyngeal nerve was also seen. The glossopharyngeal nerve was then traced supero-laterally. The styloid process was then palpated and muscles attached to it were cleared. The styloglossus muscle (SG), stylohyoid (SH), and the stylopharyngeus muscle (SP) were cleared (Figure 5).

To verify the feasibility, acceptability, and validity of the modified approach, other postgraduate students and senior residents in the department were asked to perform the dissection of the ITF and SMR using the modified approach. Feedback about the method was collected from the residents. The first-year undergraduate students (n=25) were shown the structures on both the sides of the face (one side dissected with the classical textbook method, and the other side with the modified method) and were given a questionnaire to get their comparative feedback. These students were selected randomly and were blinded for the method of dissection on either side. The questionnaire included 10 questions (Table 1). The answers to the questions were one among the two options: A - left side (traditional textbook method), and B - right side (modified method). The last question was aimed to collect feedback from the postgraduate students and senior residents.

**Table 1:** Questionnaire based upon comparison between the two ways of dissecting sub-mandibular region: Choose one option (A/B) to answer the following questions. A=Left side; B=Right side.

S. No.	Question	Option
1	Which side of dissected cadaver, you found to be better for visualizing the structures of sub-mandibular region?	A/B
2	On which side of dissected cadaver, course of lingual nerve and its relation with the surrounding structures were better seen?	A/B
3	The course of hypoglossal nerve and its relation with the loop of lingual artery was better seen on which side of dissected cadaver?	A/B
4	According to you the orientation of sub-mandibular region was better understood on which side of dissected cadaver?	A/B
5	Which dissected side of the cadaver helped in better understanding of styloid apparatus?	A/B
6	Which dissected side of the cadaver helped in better understanding of the relations of Mylohyoid muscle?	A/B
7	On which side of dissected cadaver sub-mandibular ganglion and its relations was better seen?	A/B
8	On which side of dissected cadaver structures present in infra-temporal fossa were better seen?	A/B
9	The branches of External Carotid Artery were better exposed in which side of the dissected cadaver?	A/B
10	In future which dissection approach will you prefer for dissecting the infra-temporal and sub-mandibular region?	A/B
11	Would you like to enlist the benefits and drawbacks of newer method of dissecting sub-mandibular and infra-temporal region [This question was asked only from residents].	

## Results

The structures which were better seen after dissection using the modified technique were the complete course and relations of the lingual nerve, extra-cranial course of the hypoglossal nerve and its relation with the loop of the lingual artery, nerve to mylohyoid approaching mylohyoid muscle and its branch going towards anterior belly of the digastric muscle. The mylohyoid muscle along with the superficial relations of the hyoglossus muscle, the styloid process along with the stylohyoid, styloglossus, and stylopharyngeus muscles attached to it, the anterior and posterior bellies of digastric muscle, the genioglossus muscle, and the geniohyoid muscle were easily traced. The deep lobe of the submandibular gland with the characteristic loop of the facial artery, the sublingual gland, all the branches of the external carotid artery, and the sphenomandibular ligament were clearly seen.

All of the 25 students participated in the feedback. Twenty-four (96%) students agreed that the modified approach of dissection allowed better visualisation of the structures which include lingual nerve, hypoglossal nerve, relations of the mylohyoid muscle, branches of the external carotid artery, the submandibular ganglion, relation of the lingual artery with the hypoglossal nerve, the submandibular gland with loops of the facial artery, duct of the submandibular gland with characteristic looping of the lingual nerve, and the styloid apparatus. One out of the 25 students (4%) found the modified method to be confusing due to a lack of orientation. Response to the modified method from the residents and undergraduate students were analysed and compared in light of the standard dissection book. Responses included 1) It’s an easier method for the dissector, 2) Less time consuming, 3) Less expertise required, and 4) Inferior alveolar nerve and vessels cannot be preserved (Table 2).

**Table 2:** Comparison of the students' and the residents' perceptions about the traditional textbook method and the modified method.

Dissection features	Traditional textbook method	Present method
Procedure for infra temporal region	An oblique cut applied to remove coronoid process extends from mandibular notch to anterior border of ramus where it meets the body.^5,7^	Two linear cuts at right angle applied to remove coronoid process. Transverse cut extends through the base of coronoid process followed by vertical cut from mandibular notch vertically downward intersecting transverse cut.
Steps for sub-mandibular region	Body of mandible retracted upward to expose submandibular gland, posterior belly of Digastric and Styloglossus muscle and to dissect hypogossal nerve, lingual nerve, submanidibular ganglion and duct.^5^ Body of mandible cut along the midline followed by mid sagittal sectioning of head and neck. Structures of SMR were then exposed by retracting body of mandible.^7^	Body and ramus of mandible removed to expose submandibular gland and its relations.
Better oriented	Yes	Less orientated (Require explanation for orientation).
Preservation of dissected part as museum specimen.	Possible.	Not possible
Inferior alveolar nerves and vessels	Preserved. However complete course is not seen.	Only cut edges seen.
Better visualization of deeper structures.	No	Yes
Easier.	No	Yes
Time saver.	Time consuming steps of dissection.	Need less time.
Expertise required	Yes (During deep dissection traumatic excision of superficial structures occur).	Lesser expertise required.
Sagittal section required (to see tongue muscles and deeper structures)	Yes	No

## Discussion

Cadaveric dissection has been accepted as a fundamental teaching tool in the anatomy laboratory. It facilitates students to develop theoretical and practical skills [9]. Since the infratemporal fossa and submandibular region are densely packed regions of the head and neck with irregular margins [10], many students experience difficulty in learning anatomy, especially dissection with comprehension. Understanding the topographical relations of the structures present in the ITF and SMR is pivotal for maxillofacial surgeons, otolaryngologists, and interventional radiologists to safely explore the area involved in pathology [3].

The dissection approach, as per standard anatomy books written by Romanes and Tank [8,11], describe excision of the coronoid process along an oblique line which extends from mandibular notch to the point of junction of the anterior border of the ramus of the mandible and its alveolar border (body of the mandible), followed by the removal of the outer table of the mandible to expose the inferior alveolar nerve and vessels. In the next step, these books prescribe deep dissection within the body of the mandible in situ. Standard books recommend the sagittal section of head and neck and subsequent medial approach to complete the deep dissection [8,11]. The steps of dissection described in dissector handbooks require expertise and are time consuming. In spite of this, the field of dissection does not reveal the complete course of all the important neuro-vascular structures.

In the modified approach, the excision of coronoid process is done by two incisions - Lines 1 and Line 2 (Figure 1). This removal of coronoid process avoids damage to the inferior nerve and vessels which now can be easily cleared by retracting the ramus of mandible in further local dissection. This is followed by hemi-mandibulectomy, performed by an incision immediately lateral to the site of attachment of anterior belly of digastric muscle on mandible (Line 3, Figure 1). Now the deep dissection can be performed through a lateral approach as a result sagittal sectioning of the head and neck. This is not required as in the traditional approach, whereas it results in better exposition of the regional structures. Hence, the modified approach is simpler and results in easier exploration of the structures without disturbing the regional anatomy.

The current literature features studies that highlight multiple surgical approaches to ITF and SMR [4,5,12-14]. Such approaches are guided by the disease, operative procedure, and surgeon’s expertise. However, to the best of our knowledge, none has emphasized in teaching first-year medical students the detailed anatomy of ITF and SMR. Prades et al. described a combined trans-mandibular and trans-zygomatic approach to the infratemporal fossa [14]. Their paper correlates with the current study. However, their approach did not include removal of the body of mandible as it was part of operative training for surgical management rather than in teaching undergraduate students.

Teaching anatomical relations entails elaborate dissection as described in the current article. The complementation of medical curriculum with additional disciplines like skills, professionalism, ethics, and communication has resulted in a reduction of the total number of hours allocated for cadaveric dissection. Moreover, it is desirable that students get enough exposure to get a lasting impression of this region. Based on the students’ and residents' feedback, it can be reliably concluded that dissection of the infratemporal and submandibular regions can be done using the modified approach, which is easier, needs lesser time to explore the structures placed in the deeper plane, and allows better visualization of the surgically important structures.

The modified method of dissection has certain limitations, as post-hemi-mandibulectomy, the orientation of the specimen is disturbed. Though the traditional method provides an opportunity for the students to study the mandibular course of the inferior alveolar nerve and vessels, these structures are sacrificed to allow the dissection steps in the modified method. This can be overcome by making the students show these structures before the hemi-mandibulectomy step of the modified method. The specimen dissected by this approach cannot be mounted as a museum specimen due to the absence of the mandible to provide support to the soft tissue. Hence, this approach is good for teaching in the dissection hall, but the orientation and scope of using the specimen as a museum model are sacrificed.

## Conclusions

Cadaveric dissection plays a key role in teaching and learning human anatomy. In accordance with the present medical curriculum, with its reduced number of anatomy teaching hours, we reiterate that the modified dissection approach is the superior approach to learning and understanding the anatomy of the ITF and SMR, and should be adopted for the benefit of students. The modified approach is accepted to be easier, less time-consuming, and providing a better view of the course and relations of the structures in the infratemporal and submandibular regions. Precise knowledge of anatomy of the peri-mandibular region is paramount for medical students and reconstructive surgeons. The modified dissection approach will have a vital role in the better understanding of the anatomical relations in the infratemporal and submandibular regions.
